# Morphological and functional decline of the SNc in a model of progressive parkinsonism

**DOI:** 10.1038/s41531-025-00873-9

**Published:** 2025-01-29

**Authors:** Jacob M. Muñoz, John T. Williams, Joseph J. Lebowitz

**Affiliations:** Vollum Institute, Oregon Health & Science University, Portland, OR USA

**Keywords:** Parkinson's disease, Neurophysiology, Synaptic transmission

## Abstract

The motor symptoms of Parkinson’s Disease are attributed to the degeneration of dopamine neurons in the substantia nigra pars compacta (SNc). Previous work in the MCI-Park mouse model has suggested that the loss of somatodendritic dopamine transmission predicts the development of motor deficits. In the current study, brain slices from MCI-Park mice were used to investigate dopamine signaling in the SNc prior to and through the onset of movement deficits. Electrophysiological properties were impaired by p30 and somatic volume was decreased at all time points. The D2 receptor activated potassium current evoked by quinpirole was present initially, but declined after p30. In contrast, D2-IPSCs were absent at all time points. The decrease in GPCR-mediated inhibition was met with increased spontaneous GABA_A_ signaling. Dendro-dendritic synapses are identified as an early locus of dysfunction in response to bioenergetic decline and suggest that dendritic release sites may contribute to the induction of degeneration.

## Introduction

Parkinson’s Disease (PD) is defined by gradually worsening motor symptoms resultant from the degeneration of SNc dopamine neurons. Various strategies can achieve symptomatic relief in individuals with PD, including pharmacological restoration of brain dopamine with the precursor molecule L-DOPA or deep brain stimulation^1,2^. However, a therapeutic that can slow or prevent the degeneration of SNc cells has yet to be identified^3^. Treatments for PD are also complicated by the fact that clinically identifiable symptoms do not emerge until at least 30% of dopamine cells have been lost^4^. And while the severity of motor symptoms increases throughout the duration of the lifespan following diagnosis, the loss of dopaminergic neuropil appears to plateau by ~4 years following diagnosis^5^. Thus, it has proven difficult to define a clinically relevant “prodromal” stage of PD during which dopamine cells could be targeted for neuroprotection^6^.

A major strategy to identify prodromal stages of PD is the generation of transgenic mouse models that result in a gradual loss of dopamine cells. One way to achieve this is to compromise mitochondrial function in dopamine cells, in line with evidence of mitochondrial dysfunction observed in postmortem brains from individuals with PD^7–9^. The recently generated MCI-Park mouse model selectively deletes the catalytic core of mitochondrial complex I (*ndufs2*) in dopamine cells^10^. The resultant phenotype is a loss of dopamine cells that begins in striatal axon terminals ~p30 and progresses to cell bodies in the midbrain by ~p60. Like clinical PD, MCI-Park mice exhibit gradually worsening motor symptoms that are responsive to treatment with L-DOPA, though it’s efficacy lessens through time^10^.

Initial experiments examining dopamine release in MCI-Park mice suggests that the loss of release in the midbrain occurs ~p60 and more closely tracks the development of motor symptoms than the loss of release in the striatum which is observed by ~p30^10^. However, in that work dopamine release in the midbrain was imaged using a fluorescent dopamine sensor following pre-incubation with L-DOPA, introducing the potential confound of ectopic dopamine release rather than release from dopamine neurons themselves^10,11^. Somatodendritic dopamine release, can act on several cell types including dopamine neurons themselves^12,13^. Dopamine cells express the G_I/O_ coupled D2 receptor that when activated hyperpolarizes the cell through a G-protein gated potassium (GIRK) conductance to pause firing on the order of seconds^14,15^. This inhibitory postsynaptic conductance (D2-IPSC) was previously shown to be reduced in the MitoPark model of dopamine cell loss by 6–10 weeks of age^16,17^. However, it is not known if the dopamine release in the early stages of cell decline in MCI-Park animals originates from dopamine cells or ectopic sites such as 5-HT terminals. The present study used whole cell recordings in MCI-Park mice to examine the activation of D2 receptor-mediated GIRK conductance during the earliest stages (p30-p44) of dopamine cell decline. As previously reported, dopamine cells exhibited dysregulated pacemaking and decreased HCN channel-mediated currents. Further, a decrease in the cell capacitance was correlated to an increase in input resistance, a loss of somatic volume beginning at p30, and a dramatic reduction in the dendritic arbor by p40. D2 receptor-dependent activation of GIRK conductance using exogenously applied agonists was similar to controls at p30-34, but was severely compromised by p40. However, D2-IPSCs were absent at all time points tested, suggesting somatodendritic release sites are lost during initial stages of dopamine cell decline. Conversely, GABA_B_-mediated IPSCs, which signal through the same G-protein cascade as D2Rs, were not different from age-matched controls at all time points, though an increase in spontaneous GABA release measured by GABA_A_ mediated IPSCs was observed. Taken together, these data suggest that somatodendritic dopamine transmission between SNc cells is lost before the onset of motor symptoms, and may similarly be lost during prodromal stages of PD. The changes to electrical properties and GABA-mediated inhibition may modulate dopamine cell activity to compensate for the loss of negative feedback inhibition by D2-IPSCs. Finally, while the progression of cell loss in PD is believed to begin in axon terminals and progress retrogradely to the soma and dendrites, dendritic release sites appear to be similarly sensitive to bioenergetic decline as their axonal counterparts and may be among the earliest sites of degeneration in dopamine cells.

## Results

### Intrinsic properties of dopamine neurons in MCI-Park mice

**Fig. 1:**
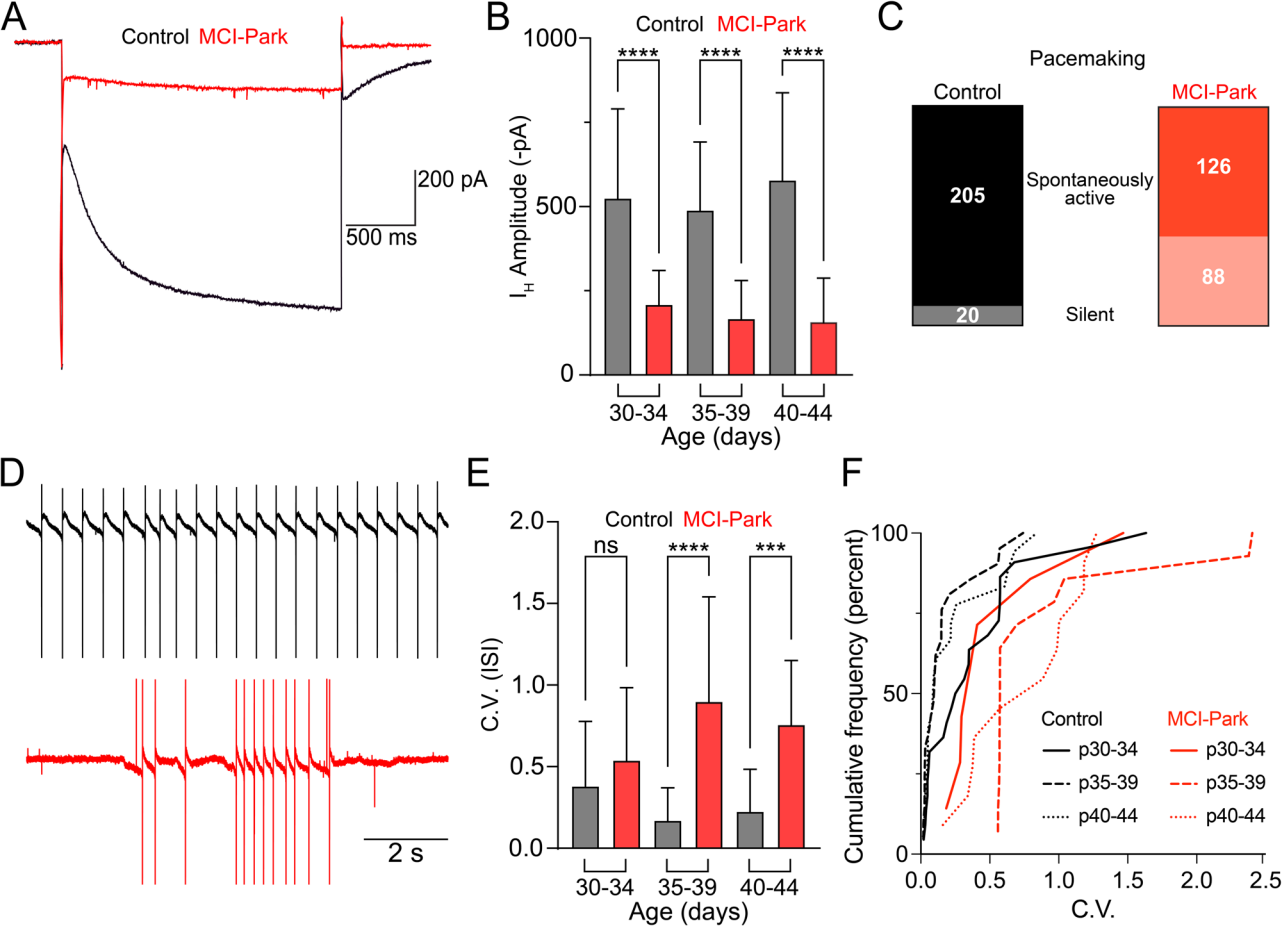
Reduced HCN-channel currents and irregular spontaneous activity in DA cells from MCI-Park mice. **A** Representative I_H_ current traces in control (black) and MCI-Park (red) induced by a 2 s, −50 mV hyperpolarizing step in 40–44 day old animals. **B** Summary data showing average I_H_ current amplitudes in control (gray) and MCI-Park (red) animals at 30–34 days (*n* = C:36/5, M:12/3; *p* < 0.0001) 35–39 days (*n* = C:43/6, M:24/4; *p* < 0.0001), and 40-44 days (*n* = C:14/2, M:14/3; *p* < 0.0001; all groups Sidak’s test following Two-way ANOVA). **C** Pooled data across age groups showing the number of cells that exhibited spontaneous activity or were silent during a 2–3 min period in cell-attached configuration prior to establishing whole-cell voltage clamp for recording experiments. **D** Representative spontaneous firing patterns from a control cell (black, top) and from an MCI-Park cell (red, bottom) recorded prior to establishing whole-cell voltage clamp. **E** Summary data of pacemaking regularity defined by the coefficient of variation (C.V) of the interspike-interval (ISI) from a subset of spontaneously active control and MCI-Park cells at 30–34 days (*n* = C:22/5, M:9/3; NS, *p* = 0.1511), 35–39 days (*n* = C:21/6, M:14/3; *p* < 0.0001), and 40–44 days (*n* = C:18/5, M:11/3; *p* = 0.0009; all groups Sidak’s test following Two-way ANOVA). **F** Cumulative probability plot of the ISI C.V. for control and MCI-Park cells by age group (n identical to **E**). Data are presented as mean + SD. n are reported as (cells/animals) for control (C) or MCI-Park (M) groups.

Initial work in the MCI-Park model identified alterations in electrophysiological properties typically used to identify dopamine neurons in the SNc^10^. The intrinsic properties of dopamine cells in MCI-Park animals and littermate controls were examined in acute ex vivo slices at three time points: p30-p34, p35-p39, and p40-p44. As reported previously, the hyperpolarization-induced inward current (I_H_) was reduced in cells from MCI-Park animals as early as p30 with MCI-Park animals averaging approximately an 80% reduction in I_H_ amplitude relative to controls (Fig. 1A, B). We also found that cells from MCI-Park animals at p35-39 and p40-44 had a greater input resistance then their control counterparts (Supplementary Fig. 1).

In slices from wild-type animals, dopamine neurons of the SNc exhibit slow (1–5 Hz) and rhythmic spontaneous firing (i.e., pacemaking). Pacemaking in MCI-Park animals and littermate controls was measured using cell-attached recordings prior to the onset of whole-cell recordings. In slices from control mice, spontaneous firing was observed in 205/225 dopamine cells (Fig. 1C). However, in slices from MCI-Park animals across all time points, only 88/214 cells exhibited any spontaneous firing prior to break-in. In cells from MCI-Park animals that did fire, the C.V. was significantly higher than control mice beginning at p35 (Fig. 1D–F). Thus, sustained pacemaking is compromised in SNc dopamine cells of MCI-Park mice as early as p30, with the firing in active cells becoming progressively more irregular through time. The data suggest that the dysregulated firing patterns are caused by multiple adaptations during bioenergetic decline including decreased subthreshold currents (such as I_H_) as well as a greater sensitivity to synaptic input owing to the change in membrane properties.

### Morphology of SNc dopamine cells in MCI-Park mice

**Fig. 2:**
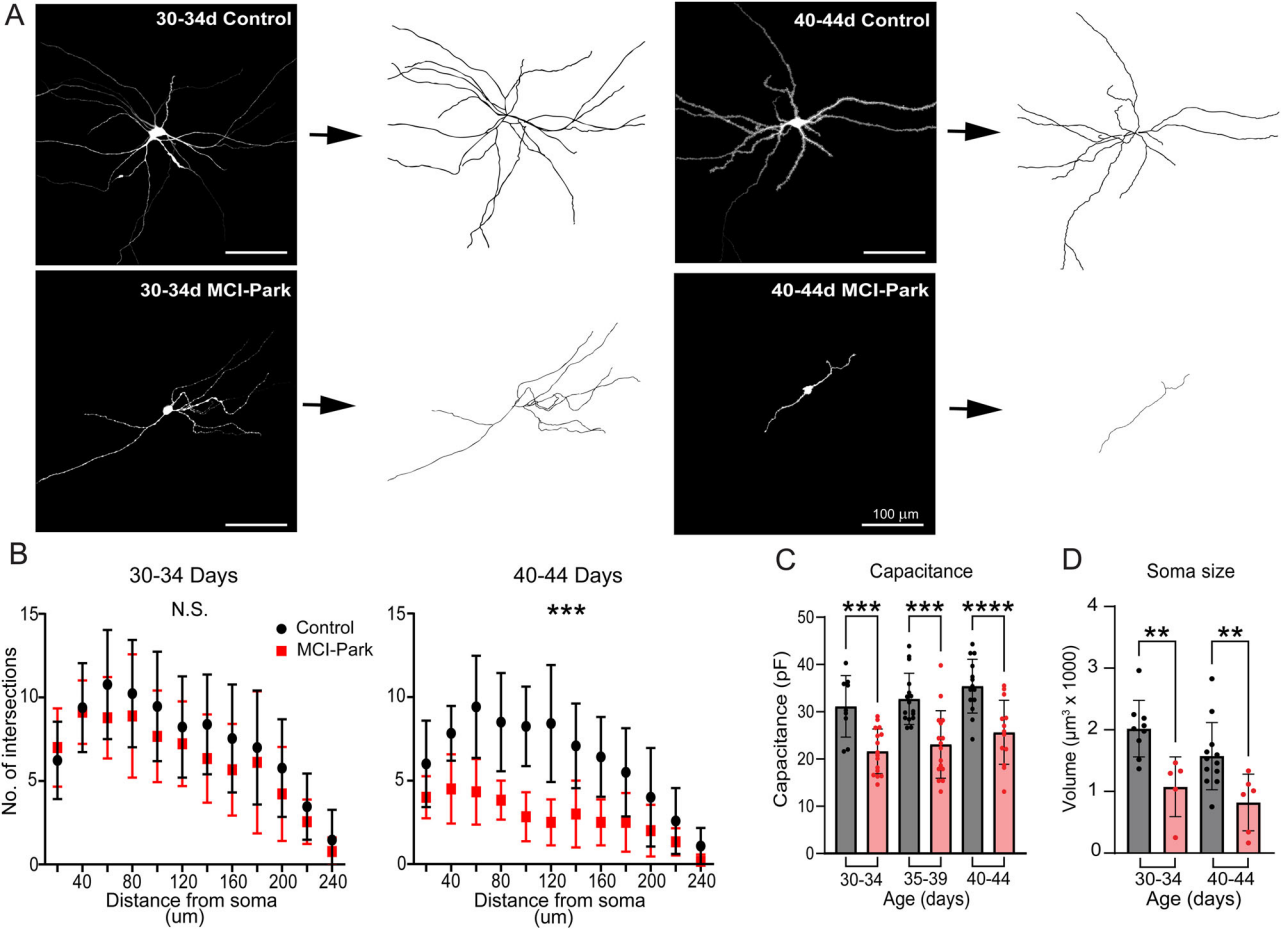
Dendritic pruning of SNc dopamine neurons in MCI-Park mice. **A** Representative single cell images (left of arrows) and skeletonized reconstructions (right of arrows) from control and MCI-Park animals. **B** Sholl analysis of reconstructed SNc dopamine neurons at p30-34 (*n* = C:13/3; M:9/3; NS effect of genotype, *p* = 0.2164, Two-Way ANOVA) and p40-44 (N = C:12/3, M:6/4, effect of genotype *p* = 0.0004, Two-way ANOVA) (**C**) Summary data comparing capacitance between control (gray) and MCI-Park (red) animals at 30–34 days (*n* = C:9/3, M:16/5; *p* = 0.0003), 35–39 days (*n* = C:16/4, M:12/4; *p* = 0.0002), and 40–44 days (*n* = C:16/6, M:11/3; *p* < 0.0001; all groups Sidak’s test following two-way ANOVA). **D** Summary data comparing somatic volume between control (gray) and MCI-Park (red) animals at 30–34 days (*n* = C:9/2, M:5/2; *p* = 0.0021), and 40–44 days (*n* = C:12/3, M:6/4; *p* = 0.0053; Uncorrected Fisher’s LSD following two-way ANOVA,). Data are presented as mean ± SD. n reported as (cells/animals) for control (C) or MCI-Park (M) groups.

Previous work in the MitoPark mouse model revealed a loss of dendritic arbor and somatic volume in late stages of dopamine cell decline^18^. This possibility was examined in early stages of dopamine cell decline in MCI-Park animals using post-hoc reconstructions of single cells filled with neurobiotin (0.05%, 10–20 min) via a patch pipette and labeled with a fluorescent streptavidin (Fig. 2A, B). There was no significant difference between genotypes at p30-34, but by p40-p44 MCI-Park animals exhibited a significant reduction of the dendritic arbor (Fig. 2B). Cell capacitance was compared between genotypes and was decreased by ~30% in MCI-Park cells relative to control cells at all time points (Fig. 2C). Reconstructions also revealed a reduction in somatic volume in MCI-Park cells at both p30-p34 and p40-p44 (Fig. 2D). The decrease in soma size and loss of dendritic complexity are consistent with the observed decrease in capacitance of MCI-Park cells, supporting the notion that dopamine neurons undergo degeneration in response to bioenergetic decline prior to overt motor symptom onset.

### Synaptic activation of D2 and GABA_B_ receptors

**Fig. 3:**
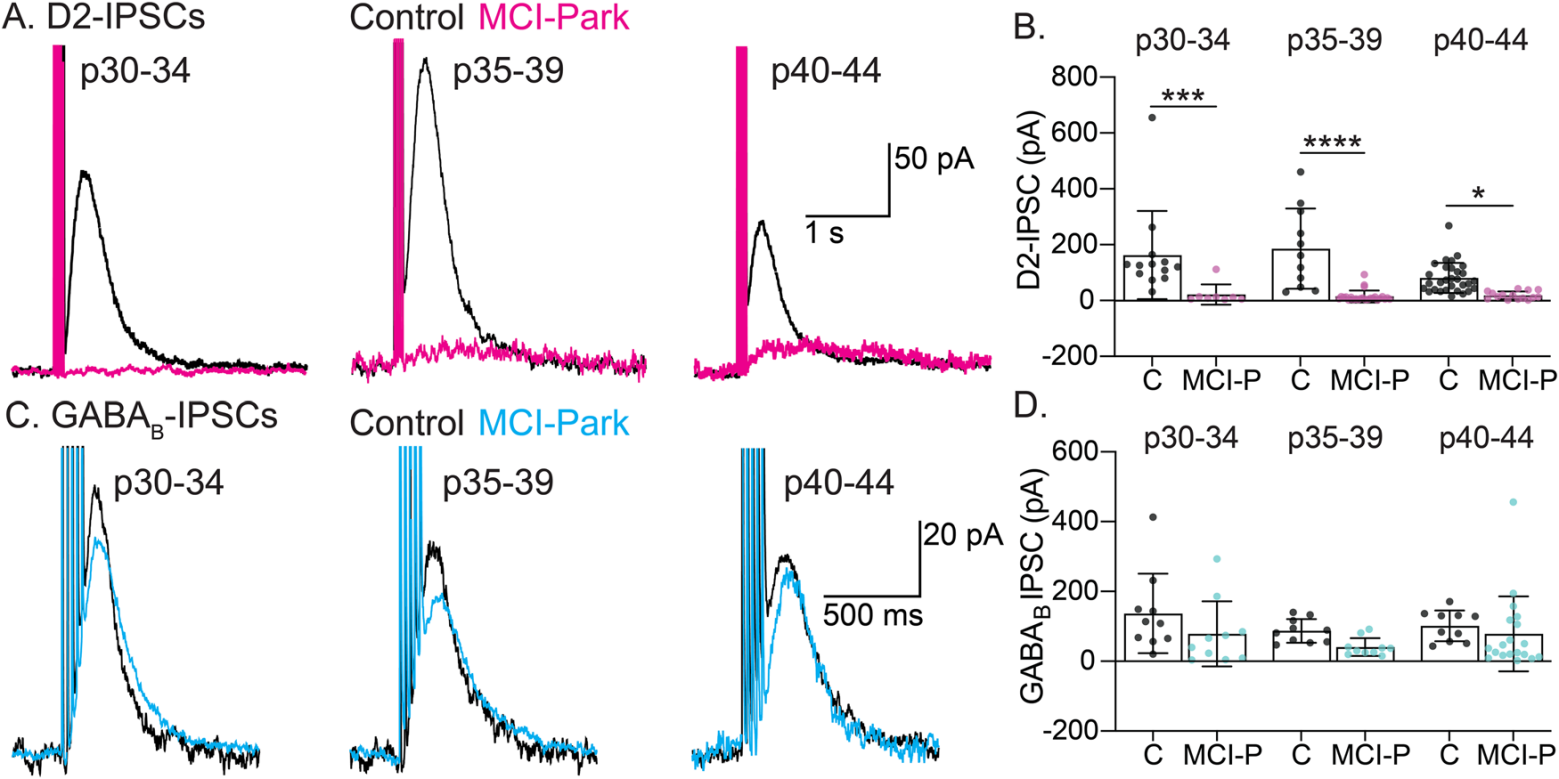
Loss of D2-mediated IPSCs and preservation of GABA_B_-mediated IPSC’s in early stages of dopamine cell decline. **A** Representative D2R-mediated IPSC’s induced by 5 electrical stimuli at 40 Hz across age groups for Control (black) and MCI-Park (magenta) cells. **B** Summary data showing the maximum amplitude of D2R-mediated IPSCs at the indicated age ranges in Control (**C**) and MCI-Park (MCI-P) animals (p30-34: *p* = 0.0001; p35-39: *p* < 0.0001; p40-44 *p* = 0.0153; n: C p30-34: 13/3, p35-39: 11/3, p40-44: 30/7; MCI-P p30-34: 8/3, p35-39: 25/6, p40-44: 15/5). **C** Representative GABA_B_-mediated IPSCs induced by 5 electrical stimuli at 40 Hz at the indicated age ranges for Control (black) and MCI-Park (cyan) animals. **D** Summary data showing the maximum amplitude of GABA_B_-mediated IPSCs at the indicated age ranges in Control (**C**) and MCI-Park (MCI-P) animals.(p30-34: *p* = 0.1206; p35-39: *p* = 0.2150; p40-44 *p* = 0.4896; n: C p30-34: 10/2, p35-39: 10/2,p40-44: 10/2; MCI-P p30-34: 10/4, p35-39: 10/2, p40-44:19/5) All comparisons made using Sidak’s test following two-way ANOVA and summary data presented as mean ± SD. n are reported as (cells/animals).

Previous work in MCI-Park mice revealed that dopamine release from the somatodendritic compartment persisted after the loss of release from terminals in the striatum^10^. Electrical stimulation in the SNc was used to examine D2-IPSCs in the SNc of MCI-Park and control animals across all time points. D2-IPSCs were readily observed in control mice from all age groups (average amplitude across control mice: 120.7pA; range: 5.1–655.2 pA). However, D2-IPSCs were absent in nearly all cells tested from MCI-Park mice by p30 (average amplitude across MCI-Park mice: 18.80 pA; range: 3.4–111.8 pA). The degree to which L-DOPA treatment restored D2-IPSCs was examined by acutely exposing slices to 10 µM L-DOPA, which has previously been shown to increase D2-IPSC amplitude^11^. Surprisingly, L-DOPA did not augment D2-IPSCs in MCI-Park slices at either p32 or p39 (Supplementary Fig. 2A, B, MCI-Park data pooled across time points). The outward whole-cell current induced by L-DOPA was also decreased in MCI-Park mice, suggesting that while functional D2 receptors were present, their signaling was reduced (Supplementary Fig. 2C). The loss of D2-IPSCs could be a result of the elimination of dendrodendritic synapses between dopamine cells (which could entail the loss of presynaptic release machinery, the elimination of postsynaptic receptor sites, and the loss of trans-synaptic organizing molecules), altered intracellular G-protein signaling, and/or decreased GIRK channel expression. To address the possible intracellular mechanisms of the loss of dopamine dependent IPSCs, synaptically evoked GABA_B_ IPSCs were examined (Fig. 3C, D). Analysis of GABA_B_-IPSCs revealed no difference between MCI-Park and controls at each matched time point. As GABA_B_-IPSCs signal through the same intracellular pathways and GIRK channels, the lack of D2-IPSCs in MCI-Park mice is therefore most likely due to a loss of dopamine and/or a loss of dendrodendritic synapses.

### Exogenous activation of D2 and GABA_B_ receptors

**Fig. 4:**
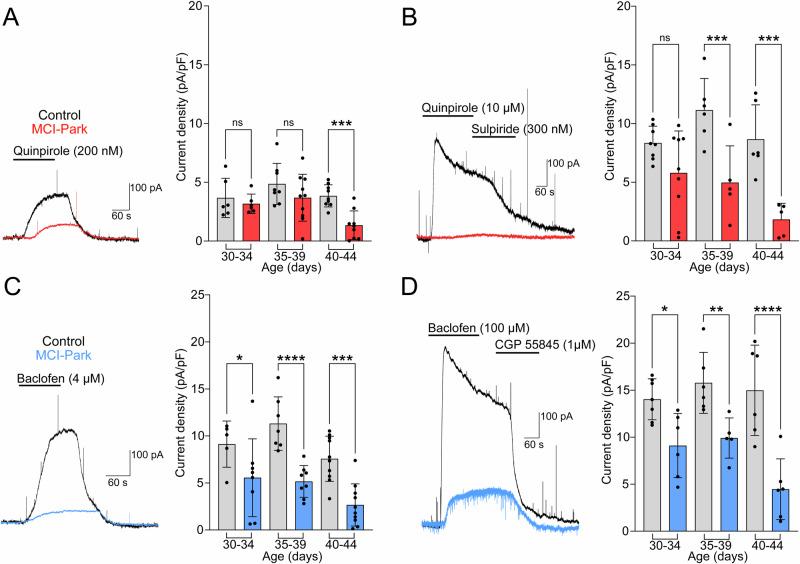
Progressive decrease in D2 and GABA-B receptor signaling in the SNc of MCI-Park mice. **A** Representative traces from p40-44 control (black) and MCI-Park (red) cells showing D2-mediated GIRK currents in response to 200 nM quinpirole and summary data of the maximum current density in control (gray) and MCI-Park (red) cells at 30–34 days (*n* = C:6/2, M:6/4; ns, *p* = 0.5660), 35–39 days (*n* = C:8/3, M:10/4; ns, *p* = 0.1041), and 40–44 days (*n* = C:9/6, M:9/3; *p* = 0.0010). **B** Representative traces from p40-44 control and MCI-Park cells in response to 10 µM quinpirole and reversal by sulpiride (300 nM) and summary data of the maximum current density in control (gray) and MCI-Park (red) at 30–34 days (n = C:8/5, M:9/6; ns, *p* = 0.0616), 35–39 days (*n* = C:6/3, M:5/3; *p* = 0.0007), and 40–44 days (*n* = C:6/4, M:5/2; *p* = 0.0002). **C** Representative traces from p40-44 control (black) and MCI-Park (blue) cells showing GABA_B_-mediated GIRK currents in response to 4 µM Baclofen and summary data from p30-34 (n = C:5/3, M:8/5; *p* = 0.0260), p35-39 (*n* = C:7/3, M:8/4; *p* < 0.0001) and p40-44 (*n* = C:10/3, M:10/3; *p* = 0.0002) animals. **D** Representative GABA_B_ mediated GIRK currents from p40-p44 control and MCI-Park cells in response to 100 µM baclofen reversed by CGP-55845 (1 µM) and summary data in control (gray) and MCI-Park (blue) cells at p30-34 days (*n* = C:7/4, M:6/5; *p* = 0.0111), p35-39 days (*n* = C:6/3, M:5/3; *p* = 0.0062), and p40-44 days (*n* = C:6/4, M:6/3; *p* < 0.0001) All groups compared using Sidak’s test following two-way ANOVA and presented as mean ± SD. n reported as (cells/animals) for control (C) or MCI-Park (M) groups.

To test D2 receptor function independent of presynaptic release sites, the outward GIRK current induced by the D2 receptor agonist quinpirole (0.2 and 10 µM) was measured. The current induced by 0.2 µM quinpirole was not different between cells from MCI-Park mice and controls at p30-p34 and p35-p39 but was reduced at p40-p44 in MCI-Park cells (Fig. 4A). At the saturating concentration of quinpirole (10 µM), cells from MCI-Park animals had reduced currents by p35-p39 with a similar progressive decrease through p40-p44 (Fig. 4B).

The GIRK current induced by GABA_B_ receptor activation was measured using the application of baclofen (4 and 100 µM). The current induced by both concentrations of baclofen was reduced in all age groups of MCI-Park animals relative to controls (Fig. 4C, D). This was surprising given the lack of reduction in GABA_B_-IPSCs, although the relative distribution of somatic and proximal or distal dendritic receptors coupled with the observed morphology changes could account for the results^19^.

### Inhibition by GABA_A_-mediated IPSCs

**Fig. 5:**
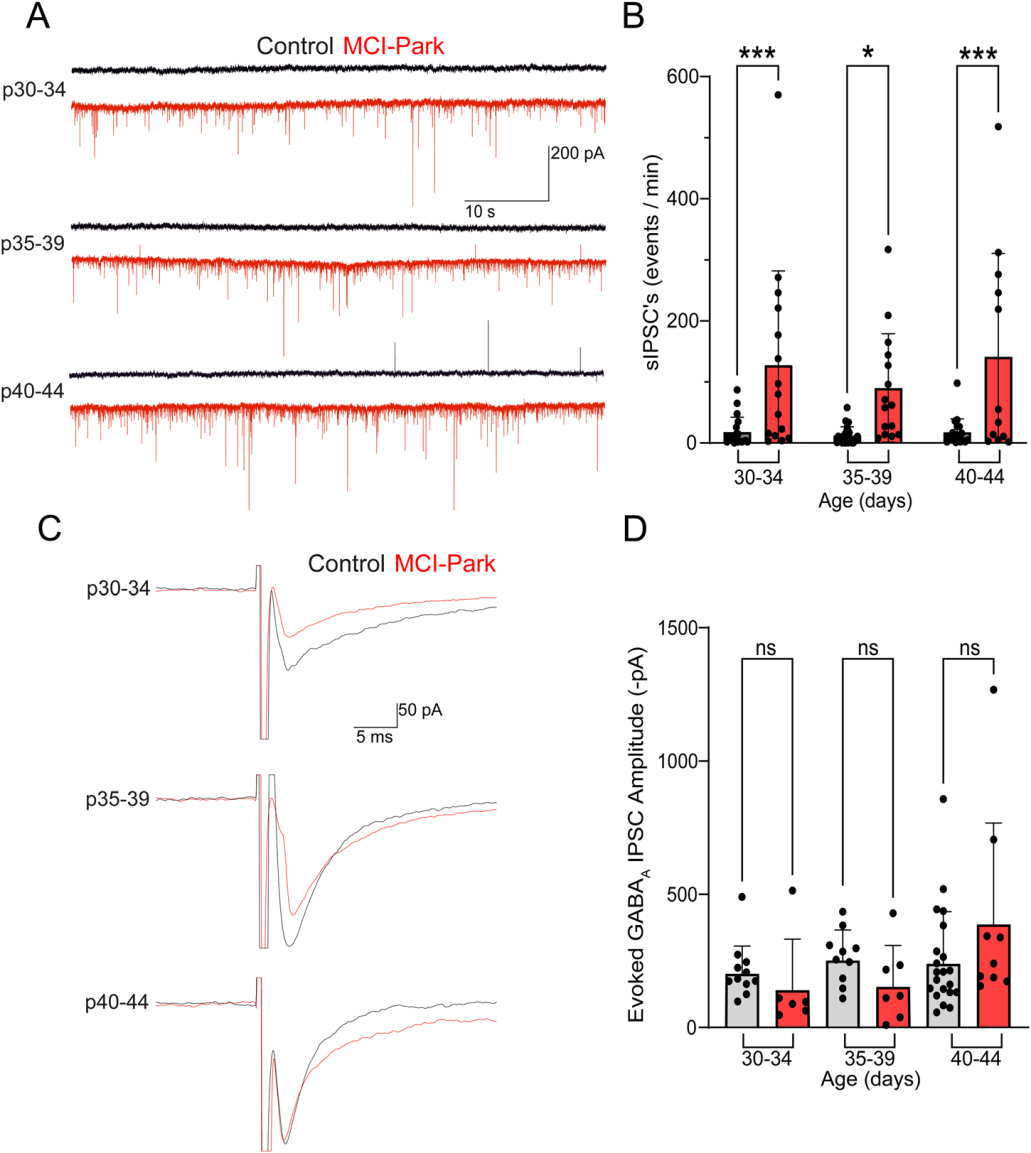
Upregulation of spontaneous GABA_A_-R signaling in SNc dopamine cells from MCI-Park mice. **A** Representative recordings of spontaneous GABA_A_ IPSCs from control (black) and MCI-Park (red) cells in the presence of nbqx for each age group. **B** Summary data of spontaneous IPSC frequencies in control (gray) or MCI-Park (red) animals at 30–34 days (*n* = C:18/3, M:15/7; *p* = 0.0008) 35–39 days (n = C:24/6, M:15/5; *p* = 0.0102). and 40–44 days (*n* = C:17/6, M:12/5; *p* = 0.0003). **C** Representative recordings of evoked GABA_A_ IPSCs in control and MCI-Park cells across age groups. **D** Summary data of the amplitude (inverted) of evoked GABA_A_ IPSCs in control (gray) and MCI-Park (red) cells at 30–34 days (*n* = C:11/4, M:6/3; NS, *p* = 0.5464), 35–39 days (n = C:10/4, M:7/4; ns, *p* = 0.3118), and 40–44 days (*n* = C:21/10, M:9/4; ns, *p* = 0.0648). All groups compared using Sidak’s test following two-way ANOVA; data in (**B**) are mean ± SD, data in (**D**) are mean + SD. n reported as (cells/animals) for control (C) or MCI-Park (M) groups.

A marked increase in spontaneous synaptic currents was frequently observed in recordings from MCI-Park cells relative to those in control cells. These spontaneous currents were not blocked by the AMPA receptor antagonist NBQX, but were completely blocked by the GABA_A_ receptor antagonist gabazine (Supplementary Fig. 3). Spontaneous IPSCs were not action potential dependent as there was no change in frequency following application of the voltage-gated sodium channel blocker tetrodotoxin (TTX, Supplementary Fig. 3). The frequency of GABA_A_ mediated sIPSCs in the presence of NBQX was increased in MCI-Park cells at all time points relative to control cells (Fig. 5A, B). In contrast, evoked GABA_A_ mediated IPSCs in MCI-Park cells were not different than those measured in control cells at any timepoint (Fig. 5C, D). The selective change in spontaneous IPSCs by p30 suggests that the pathways mediating spontaneous release of GABA are rapidly altered by the loss of brain dopamine. These changes may represent a homeostatic adaptation to restore normal levels of inhibition following the loss of inhibition by synaptically released dopamine, as has been suggested by increased GABA levels observed in the MitoPark mouse model^20^.

## Discussion

Multiple lines of evidence in animal models have identified mitochondrial dysfunction (and subsequent loss of bioenergetic capacity) as a causative factor in parkinsonian-like neurodegeneration^7,21,22^. This is supported by the induction of a parkinsonian movement disorder in humans following the ingestion of the mitochondrial toxin MPTP, as well as evidence of mitochondrial DNA damage and dysfunction in postmortem tissue from individuals diagnosed with Parkinson’s disease^8,9,23,24^. Accordingly, animal models of mitochondrial insult in dopamine cells have become a prominent means to examine the pathophysiological decline of dopamine cells preceding and during degeneration. MCI-Park mice lose mitochondrial oxidative phosphorylation in dopamine cells by ~ p20 leading to a gradual loss of SNc dopamine neurons and the development of L-DOPA responsive movement deficits^10^. The current study in MCI-Park animals examines dopamine cell physiology in neurons that are in bioenergetic decline, but prior to cell death, potentially modeling the state of dopamine cells during a prodromal phase of Parkinson’s disease.

Progressive changes in the somatodendritic morphology and basal electrophysiological properties of SNc DA cells were observed during the p30-p44 timeframe (Figs. 1, 2). GPCR (D2 and GABA_B_) signaling induced by exogenous agonist was reduced, but persisted, in MCI-Park mice at early time points and gradually decreased through the time course of examination (Fig. 4). Conversely, D2-mediated IPSCs that rely on endogenous dopamine release from the somatodendritic compartment were completely absent at the earliest time point measured, while GABA_B_-mediated IPSCs were preserved through the duration of experiments (Fig. 3). The current results add to the characterization of the MCI-Park mouse model and provide insight into early dysfunction of SNc dopamine cells during bioenergetic decline.

One striking finding from this work is the rapid loss of somatic volume and dendritic complexity in MCI-Park mice (Fig. 2). In contrast to data from the MitoPark mouse model, where effects are not observed until animals are 16–20 weeks old, dopamine cells from MCI-Park animals exhibited significant decreases in somatic volume by p30 and pruning of the dendritic arbor by p40^16,18^. While this may first seem at odds with the traditionally held view of axon-first degeneration, it may be explained by the fact that release sites at dendrodendritic synapses relies on similar molecular machinery as striatal terminals, and therefore likely imposes a comparable energetic burden on the cell^25–28^. It is also likely that the loss of dendritic neuropil may explain the observed discrepancy between preserved GABA_B_-mediated IPSCs while currents induced by exogenous agonists were decreased (Figs. 3, 4); while peri-somatic electrical stimulation triggers GABA release that predominantly activates receptors on proximal dendrites, exogenous agonism activates all receptors including those on distal dendrites and is therefore more sensitive to the loss of dendritic surface area. The peri-somatic GABA_B_-mediated IPSCs were used here as a measure of G-protein and GIRK signaling capacity, and the lack of change in this current suggests a preservation of both intracellular signaling competency and of release sites that are not subject to the mitochondrial insult. However, further work examining inhibition by GABA_B_ receptors is warranted, particularly at dendritic processes projecting to the SNr where an enrichment of GABA_B_ conductance has been reported^19^.

Previous work in the MCI-Park model found a relative preservation of somatodendritic dopamine release using a fluorescent sensor expressed on multiple neuronal subtypes in the ventral midbrain following a one-hour incubation of slices with L-DOPA^10^. In contrast, somatodendendritic release of dopamine as measured by D2-IPSCs is essentially absent at all time points in the current study, including following acute (10 min) treatment with L-DOPA (Fig. 3, Supplementary Fig. 2). The present results suggest that the anatomical sites that underlie D2-IPSCs are dramatically reduced by p30, leading to the loss of the electrophysiological readout of dopamine release. As D2-IPSCs only measure dopamine release that activates D2 receptors at dendrodendritic synapses, it is likely that the energetic demand at these sites leads to early dysfunction and elimination^13,29^. In contrast, fluorescent sensors are expressed diffusely across the plasma membrane and can therefore be activated by dopamine release that would not similarly activate synaptic D2 receptors. It is possible that release from sites distinct from dendrodendritic synapses, including spontaneous release, is sufficient to sustain movement behaviors. This is supported by recent work showing no changes to movement in mice lacking the active zone organizing protein RIM, which lose phasic dopamine release from both dendrites and terminals but retain spontaneous release^30^. Given the disparate results, more work regarding the role of dopamine signaling at sites other than dopamine cells in the ventral midbrain is required to understand its role in the onset of movement deficits.

Taken together the current work reveals rapid changes to dopamine cell function and morphology in response to bioenergetic decline. It is critical to note that the age range of MCI-Park animals used in the current study was chosen to represent a period where cognitive and fine-motor deficits are present, but prior to changes in overall locomotion^10^. However, the near total absence of D2-IPSCs suggest that dopamine transmission between SNc cells themselves is unlikely to underlie a compensatory adaptation that sustains movement during this period. What appears more likely is that changes to basal ganglia circuity downstream of the SNc and changes in afferent input to SNc cells compensate for the lack of dopamine and sustain movement in early stages of cell loss. This is in line with previous works that attribute a role to changes in GABA signaling in the clinical progression of PD, as well as the hypothesis that altered GABA signaling in the SNr may delay the onset of motor symptoms following striatal dopamine loss in MCI-Park mice^10,31–34^. Given the speed of degenerative decline in dopamine cells relative to the progression of motor deficits, future studies in MCI-Park mice should examine the potential of therapeutics that sustain compensatory changes in GABA signaling following dopamine depletion to modify disease and symptom progression.

## Methods

### Animals

All animals were housed and maintained at Oregon Health and Science University and all housing, husbandry, and experimental procedures were in accordance with an approved OHSU IUCAC protocol and the NIH’s Guide for the Care and Use of Laboratory Animals. MCI-Park mice (RRID:IMSR_JAX:036313) were generated as previously described by crossing DAT-Cre mice (RRID:IMSR_JAX006660) with mice carrying floxed alleles for *ndufs2* (RRID:IMSR_JAX:036313)^10,35,36^. Original breeding pairs were obtained from the Surmeier group at Northwestern University. All breeding pairs consisted of a male heterozygous for both Cre recombinase and the floxed *ndufs2* allele *(slc6a3-Cre*^*+/−*^; *flox-ndufs2*^+/−^) and a female homozygous for the floxed *ndufs2* allele (*slc6a3-Cre*^*-/-*^; *flox-ndufs2*^+/+^). Experimental mice (MCI-Park mice) were heterozygous for Cre recombinase and homozygous for the floxed *ndufs2* allele (*slc6a3-Cre*^*+/-*^; *flox-ndufs2*^+/+^). Control mice were heterozygous or homozygous for the *ndufs2* floxed allele with no Cre expression (*slc6a3-Cre*^*-/-*^; *flox-ndufs2*^+/-^ or *flox-ndufs2*^+/+^). All data was collected from males and females aged 30-44 days. Animals were maintained on a 12-h light cycle with 24/7 access to standard mouse chow. Litters received nutritional supplementation with moistened high-fat chow (PicoLab Mouse Diet 20, LabDiet Cat# 5058) provided daily beginning at the time of weaning (p21) until p26, and a nutrient-rich gel (DietGel Boost; ClearH_2_O, Westbrook, ME) provided every other day beginning at the time of weaning until being used for experiments. Runts or animals that appeared to be in poor health at the time of weaning were euthanized using CO_2_ in accordance with approved OHSU IACUC policies.

### Slice preparation

Animals were deeply anesthetized with isoflurane before rapid decapitation and removal of the brain into warmed (~35 °C) Krebs solution containing (in mM): 126 NaCl, 2.5 KCl, 1.2 MgCl_2_, 2.4 CaCl_2_, 1.2 NaH_2_PO_4_, 21.4 NaHCO_3_, and 11.1 Dextrose. Horizontal slices (222 µm) containing the ventral midbrain were prepared using a vibratome (Leica VT1000S). Slices were cut in the same Krebs solution as above with constant carbogen bubbling (95% O_2_, 5% CO_2_). Slices were allowed to recover for ≥30 min before experiments. All Krebs solution for extraction, cutting, and recovery contained MK-801 (10 µM) to prevent NMDA-mediated excitotoxicity.

### Electrophysiology

Slices were hemisected down the midline and one side was transferred to a recording chamber under continuous perfusion (2–3 ml/min) of carbogen bubbled Krebs solution. Recordings were obtained in whole-cell voltage clamp with glass microelectrodes (World Precision Instruments) with an open-tip resistance of 1.1–1.6 MOhm. Internal solutions for recording varied by experiment and are listed under the specific experiment’s subheading. SNc dopamine neurons from control mice were identified by their morphology and position lateral to the medial terminal nucleus of the accessory optic tract. Owing to the loss of typical morphological and electrophysiological properties, initial recordings in cells from MCI-Park animals were verified to be from dopaminergic neurons by the inclusion of neurobiotin (0.05%, Vector Labs, Cat#: SP-1120-50) in the internal solution and post-hoc labeling of neurobiotin and immunolabeling of tyrosine hydroxylase. Pacemaking was recorded in cell-attached configuration for ≥1 min prior to break in. Basal electrical properties were measured and monitored following break-in using the average of twenty 5 mV pulses (10 ms pulse, sampled at 10 kHz) and cells with a series resistance ≥14 MOhm, or with a change in series resistance ≥50% during the recording period were excluded from analysis. Electrophysiology experiments were conducted, recorded, and analyzed in Axograph with the exception of experiments measuring steady-state currents following exogenous agonist superfusion (Fig. 4) which were collected and analyzed in Labchart (ADInstruments). All recordings were collected at 1 kHz using an Axopatch 200B (Axon Instruments) while holding cells at –55 mV. HCN currents (I_H_) were measured shortly after break-in by applying a −50 mV voltage step for 2 s.

### D2 and GABA-B receptor function

Total receptor signaling was measured by agonist superfusion at least 10 min after establishing whole cell voltage clamp configuration with an internal solution containing (in mM): 100 mM K-methanesulfonate, 20 NaCl, 1.5 MgCl2, 10 HEPES (K), 2 Mg-ATP, 0.3 Na-GTP, 10 Phosphocreatine, and 0.1 EGTA. D2 receptor function was measured using quinpirole at 200 nM (EC_50_) and 10 µM (saturating) concentrations. GABA_B_ receptor function was measured using baclofen at 4 µM (EC_50_) and 100 µM (saturating) concentrations. All currents were measured as the maximum steady-state value prior to the onset of receptor desensitization.

### Evoked D2 and GABA-B receptor IPSC’s

D2 and GABA_B_ mediated IPSC’s were evoked with extracellular electrical stimulation (5 stimuli, 40 Hz, 200 µA) using a monopolar electrode within 75–100 µm of the cell being recorded from. Evoked IPSC’s were recorded with a high Ca^2+^-buffering internal solution containing (in mM): 100 K-methanesulfonate 20 NaCl, 1.5 MgCl2, 10 HEPES (K), 2 Mg-ATP, 0.3 Na-GTP, 10 Phosphocreatine, and 10 BAPTA (4 K). IPSCs were recorded once per minute and analyzed and presented data are the average of 3–5 sweeps. All Krebs solution for IPSCs contained Picrotoxin (100 µM) and NBQX (10 µM); D2-IPSCs were recorded in the additional presence of CGP-55845 (300 nM), while GABA_B_ IPSCs were recorded in the additional presence of sulpiride (600 nM). L-DOPA was made fresh daily and perfused onto slices in Krebs for a period of 10 min, with a 15 min washout. The measured IPSC was taken as the average of 3–5 consecutive sweeps during which the maximum increase in amplitude was observed.

### Spontaneous and Evoked GABA-A IPSC’s

Spontaneous GABA-A mediated IPSC’S were recorded in uninterrupted minute-long sweeps and identified using Axograph’s spontaneous event detection program. Internal solution for spontaneous IPSC experiments was either identical to the high Ca^2+^ buffering solution to evoke D2 and GABA_B_ IPSCs or used a solution containing (in mM): 50 K-methanesulfonate, 50 KCl, 20 NaCl, 1.5 MgCl2, 5 HEPES(K), 2 Mg-ATP, 0.3 Na-GTP, 10 phosphocreatine, and 10 BAPTA (4 K). No difference in spontaneous event frequency was observed between internal solutions and data was pooled for analysis. Frequency of events for all experiments were measured before and after application of NBQX (2 μM). A subset of experiments (Supplementary Fig. 3) confirmed that IPSCs were mediated by GABA_A_ receptors by application of SR-95531 (gabazine, 10 µM) and that they were action potential independent via application of tetrodotoxin (TTX, 1 µM). Evoked GABA_A_ IPSC’S were stimulated with a monopolar electrode placed ~100 µm from the recorded cell using 70–150 µA dependent on the threshold required to produce a stable IPSC. All analyzed and presented data for evoked IPSCs are the average of three sweeps.

### Immunofluorescence

Cells filled with neurobiotin (Vector Labs) via the patch pipette (0.05%, 10–20 min) were allowed to rest for ≥20 min following removal of the pipette to allow for diffusion of the tracer throughout the cell. Slices were then fixed in 4% Paraformaldehyde (Ted Pella) in phosphate buffered saline (PBS) overnight at 4 °C. Following fixation, slices were washed in PBS at room temperature. All washes were repeated 3 times for 15 min each. Blocking and permeabilization were conducted in 10% Normal Goat Serum (NGS, Southern Biotech Cat# 0060-01) and .5% triton x-100 with the addition of streptavidin conjugated to alexafluor-594 (1:500, ThermoFisher Cat#: S32356). Slices were washed and mounted on Superfrost+ slides (Fisher Scientific) with Fluoromount-G mounting media (Southern Biotech). A subset of slices were incubated in 5% NGS and .1% triton with a primary antibody against tyrosine hydroxylase antibody (1:500, Abcam Cat #: ab76442) overnight at 4 °C following blocking and permeabilization. Following primary incubation, slices were washed and incubated with a goat-anti-chicken AlexaFluor-488 secondary antibody (1:500, ThermoFisher, Cat#: A-11039) for one hour at room temperature in 5% NGS and .1% triton. Slices were washed three times for ≥20 min each prior to mounting.

### Imaging and sholl analysis

Confocal images were obtained with a Zeiss LSM 980 with Airyscan2 at OHSU’s Advanced Light Microscopy Core. Z- stacks were generated with a 424 × 424 µm field of view centered on the cell soma and the upper and lower boundaries visually set to capture the entirety of the dendritic arbor. Neurons filled with neurobiotin were reconstructed with ImageJ’s neuroanatomy plugin Simple Neurite Tracer^37^. Images were traced using the program’s manually assisted algorithm search before subsequent Sholl analysis. Sholl analysis parameters were set with an initial radius of 20 µm from the center of the cell soma and the number of intersections were recorded at increasing increments of 20 µm. Somatic volume was calculated by reconstructing solely the somatic compartment and analyzed using Fiji’s 3D object-counter volume measurement.

### Statistical analysis

Statistical analysis of data was conducted using Graphpad Prism and described. Data is presented as the mean ± standard deviation. Grouped data were analyzed with two-way ANOVA followed by Sidak’s multiple comparison test unless otherwise noted in corresponding figure legend. Data were tested for normality using the Shapiro-Wilk test where applicable. Sizes of experimental groups were not predetermined, and no outliers were removed from the data sets.

## Supplementary information

Supplementary Information

## Data Availability

Original data will be made available upon reasonable request to the corresponding author (lebowitz@ohsu.edu).
