# Supplementary material for: Morphological and functional decline of the SNc in a model of progressive parkinsonism
Source: NPJ Parkinsons Dis. 2025 Jan 29;11:24. doi: 10.1038/s41531-025-00873-9 (PMC11775090; doi:10.1038/s41531-025-00873-9)
Supplement: Supplementary file 1 — Supplementary Information [file 41531_2025_873_MOESM1_ESM.docx]

**
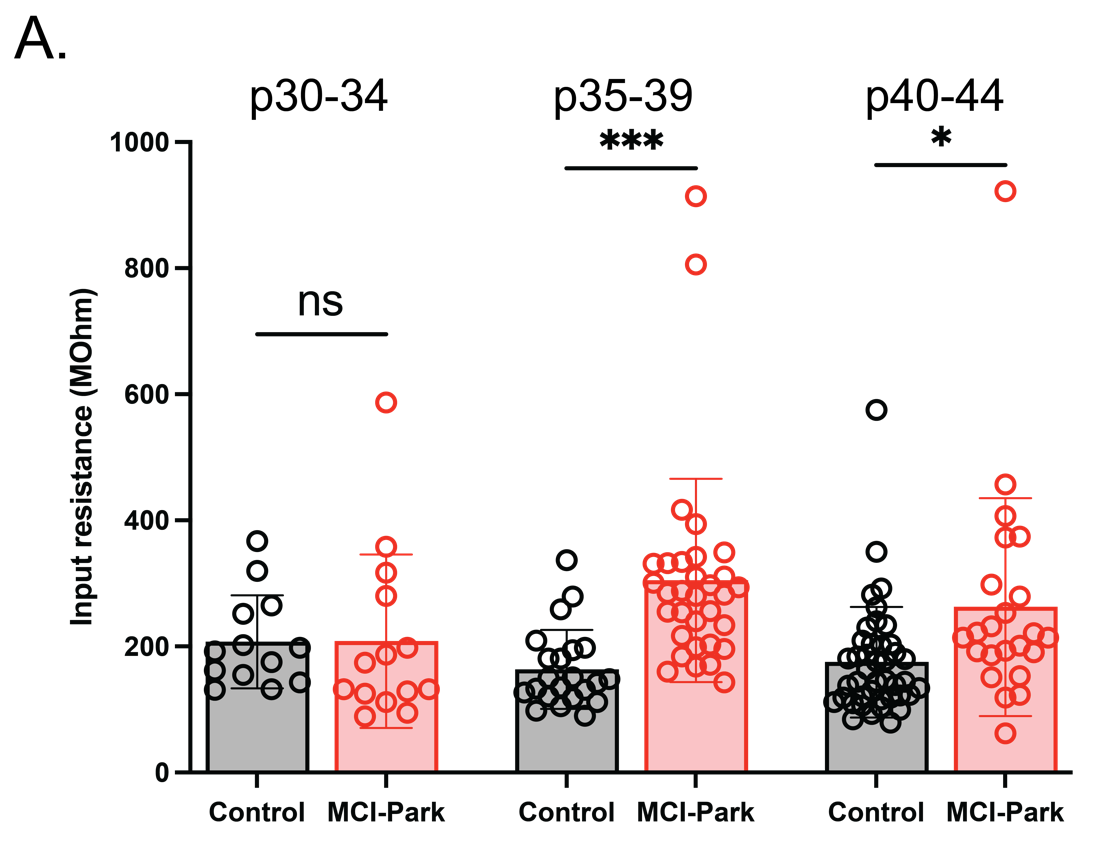
**

**Supplemental Figure 1. Increased input resistance in dopamine cells from MCI-Park animals. A.** Summary data of cellular input resistance measured using a 5 mV depolarizing step immediately after establishing whole cell configuration. Cells from MCI-Park animals resembled control cells at p30-34 (ns, p > 0.9999), but showed an increase in input resistance at p35-39 (p = 0.0002) and p40-44 (p = 0.0227). n (cells/animals) – control p30-34: 13/3, MCI-Park p30-34: 14/5; control p35-39: 22/5, MCI-Park p35-39: 32/7; control p40-44: 42/9, MCI-Park p40-44: 23/6. All comparisons made with Sidak’s test following two-way ANOVA. Data are presented as mean ± SD


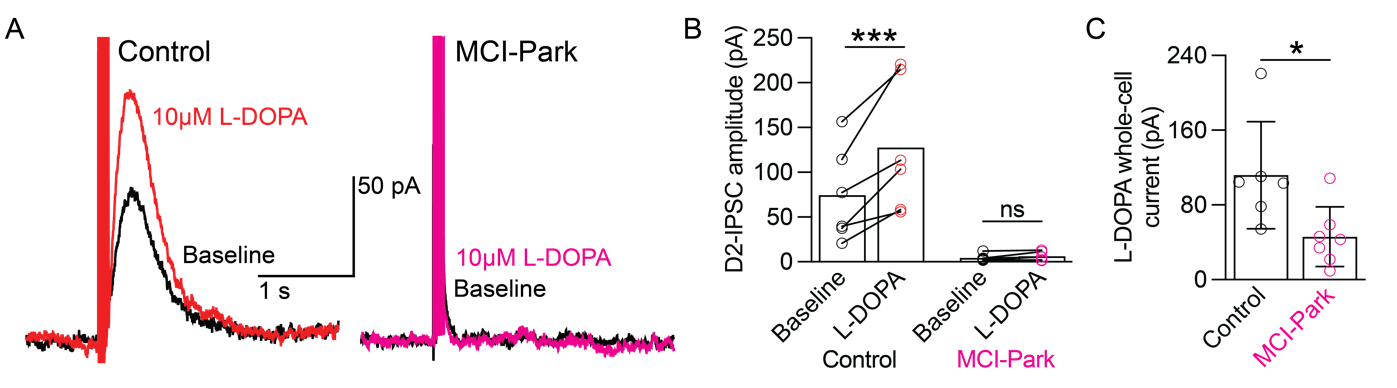


**Supplemental Figure 2. Acute L-DOPA treatment does not restore D2-IPSCs in MCI-Park slices. A.** D2-IPSCs recorded before (baseline, black), and after 10 minutes of exposure to 10µM L-DOPA (red, control; magenta, MCI-Park). **B.** Summary data of D2-IPSC amplitudes before and after 10 minutes of exposure to 10µM L-DOPA. Control cells exhibit an increase in amplitude, while only one cell of seven showed an L-DOPA induced increase, though it did not restore D2-IPSC amplitude to the level of control cells. (control baseline: 74.3 ± 52.5 pA, control L-DOPA: 127.7±73.4 pA, p = 0.0001; MCI-Park baseline: 4.3±3.5, MCI-Park L-DOPA: 6.3±4.4, ns; Sidak’s test following two-way ANOVA) **C.** Amplitude of the maximum whole-cell current measured during L-DOPA exposure. (control: 111.9±57.3 pA, MCI-Park: 45.99±32.0 pA; p=0.0240; unpaired t test). n (cells/animals) – control:6/2, MCI-Park: 7/2. Data are presented as mean ± SD


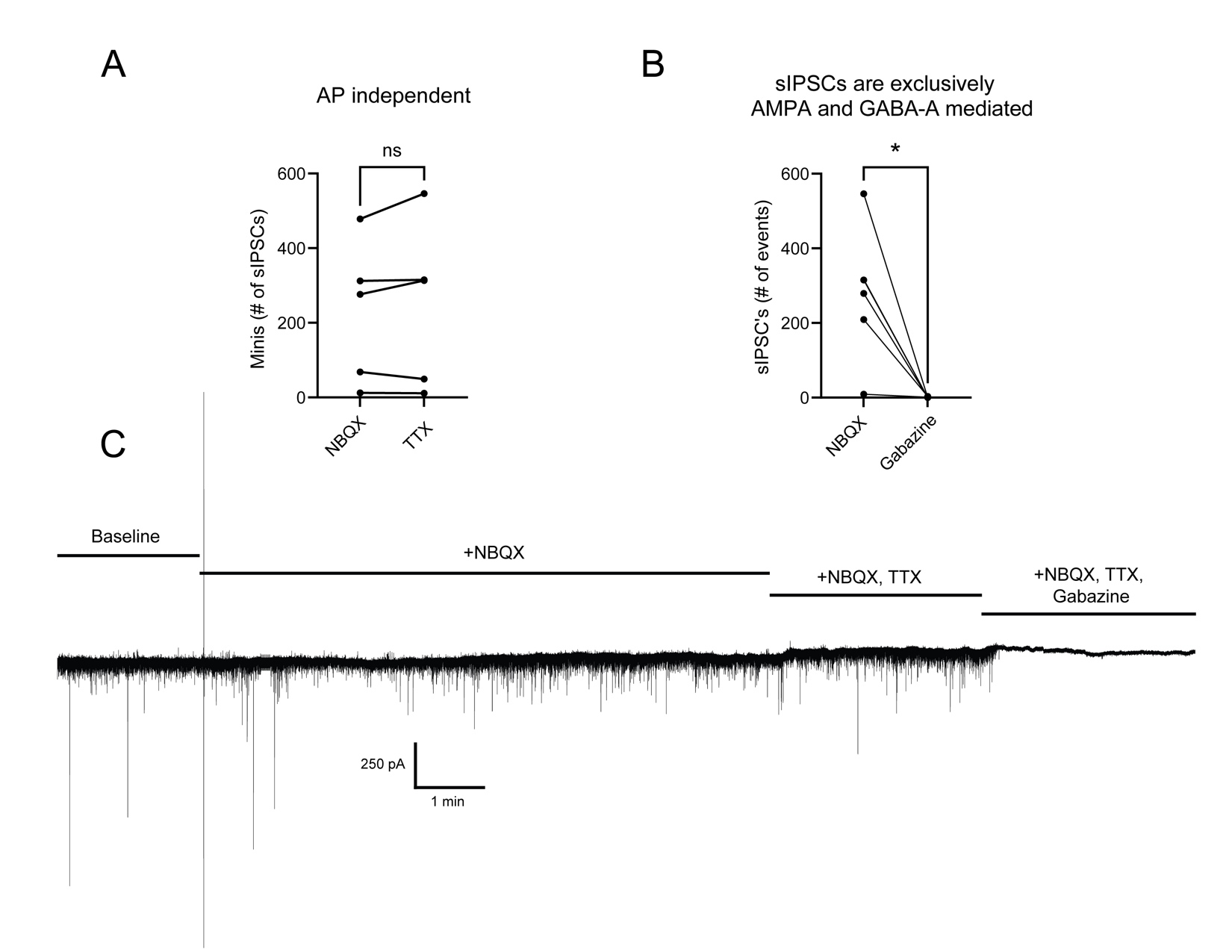


**Supplemental figure 3. Spontaneous currents in MCI-Park mice are GABA-A mediated and action potential independent** (A) Summary data of the number of sIPSCs in one minute recorded in nbqx, followed by TTX to block action potential dependent signaling (ns, p = 0.32, paired two-tailed t-test; n = 5 cells / 5 animals). (B) Summary data of the quantity of sIPSCs per minute in the presence of nbqx followed by gabazine confirming the identity of spontaneous as GABA_A_ receptor mediated (p = .0312, two-tailed Wilcoxon matched-pairs signed rank test; n = 5 cells / 4 animals). C. Representative trace from an MCI-Park cell showing sIPSCs before drug (baseline), during AMPA receptor blockade (NBQX), during blockade of AP’s (NBQX,TTX), and finally the absence of sIPSCs following GABA_A_ receptor blockade (NBQX, TTX, Gabazine)
